# A Quantitative Imaging Biomarker Supporting Radiological Assessment of Hippocampal Sclerosis Derived From Deep Learning-Based Segmentation of T1w-MRI

**DOI:** 10.3389/fneur.2022.812432

**Published:** 2022-02-18

**Authors:** Michael Rebsamen, Piotr Radojewski, Richard McKinley, Mauricio Reyes, Roland Wiest, Christian Rummel

**Affiliations:** ^1^Support Center for Advanced Neuroimaging (SCAN), University Institute of Diagnostic and Interventional Neuroradiology, Inselspital, Bern University Hospital, University of Bern, Bern, Switzerland; ^2^Graduate School for Cellular and Biomedical Sciences, University of Bern, Bern, Switzerland; ^3^Swiss Institute for Translational and Entrepreneurial Medicine, sitem-insel, Bern, Switzerland; ^4^ARTORG Center for Biomedical Research, University of Bern, Bern, Switzerland

**Keywords:** hippocampal sclerosis, epilepsy, MRI, segmentation, deep learning, brain morphometry, hippocampus

## Abstract

**Purpose:**

Hippocampal volumetry is an important biomarker to quantify atrophy in patients with mesial temporal lobe epilepsy. We investigate the sensitivity of automated segmentation methods to support radiological assessments of hippocampal sclerosis (HS). Results from FreeSurfer and FSL-FIRST are contrasted to a deep learning (DL)-based segmentation method.

**Materials and Methods:**

We used T1-weighted MRI scans from 105 patients with epilepsy and 354 healthy controls. FreeSurfer, FSL, and a DL-based method were applied for brain anatomy segmentation. We calculated effect sizes (Cohen's *d*) between left/right HS and healthy controls based on the asymmetry of hippocampal volumes. Additionally, we derived 14 shape features from the segmentations and determined the most discriminating feature to identify patients with hippocampal sclerosis by a support vector machine (SVM).

**Results:**

Deep learning-based segmentation of the hippocampus was the most sensitive to detecting HS. The effect sizes of the volume asymmetries were larger with the DL-based segmentations (HS left *d*= −4.2, right = 4.2) than with FreeSurfer (left= −3.1, right = 3.7) and FSL (left= −2.3, right = 2.5). For the classification based on the shape features, the surface-to-volume ratio was identified as the most important feature. Its absolute asymmetry yielded a higher area under the curve (AUC) for the deep learning-based segmentation (AUC = 0.87) than for FreeSurfer (0.85) and FSL (0.78) to dichotomize HS from other epilepsy cases. The robustness estimated from repeated scans was statistically significantly higher with DL than all other methods.

**Conclusion:**

Our findings suggest that deep learning-based segmentation methods yield a higher sensitivity to quantify hippocampal sclerosis than atlas-based methods and derived shape features are more robust. We propose an increased asymmetry in the surface-to-volume ratio of the hippocampus as an easy-to-interpret quantitative imaging biomarker for HS.

## 1. Introduction

Magnetic resonance imaging (MRI) is the key element in diagnosing structural lesions in epilepsy (1). High-resolution MRI, preferably with 3 Tesla (3T) including three-dimensional non-contrast T1-weighted (T1w) imaging suitable for automated postprocessing, is part of today's protocol recommendations (2, 3). In mesial temporal lobe epilepsy (mTLE), hippocampal sclerosis (HS) is the most common pathology (4). Its characteristic neuronal loss and gliosis manifesting as volume loss and increased T2 signal intensities (5) make MRI an essential clinical tool for the differential diagnosis in TLE. While HS in advanced stages is usually reliably identified in epilepsy specific MRI by experts (6), the challenge remains putative in non-lesional (MRI negative) patients in an early stage (7). Quantitative hippocampal volumetry is already recommended for patients with TLE, who were considered for epilepsy surgery (8). For clinical assessment, manual segmentations are recommended (9), a labor-intensive task requiring specific training to achieve good inter-rater agreement (10).

In this study, we selected two of the most frequently used freely available morphometry tools (11) to segment deep gray matter structures, FreeSurfer (FS) (12, 13) including segmentation of hippocampal subfields (FS-SF) (14) and FSL-FIRST (15), and contrasted the results to a deep learning (DL)-based segmentation (16).

Deep learning-based methods have been shown to outperform atlas-based methods for neuroanatomy segmentation (17–20). Convolutional neural network (CNN) architectures have also been successfully used specifically to segment the hippocampus (21–24).

In the largest morphometry study on epilepsy to date by the ENIGMA-Epilepsy group (25), volume loss of the ipsilateral hippocampus was the most pronounced effect in lesional patients with TLE (26, 27). Inter-hemispheric asymmetries of brain structures are not correlated to age in healthy conditions, i.e., are usually small and remain stable across large age ranges (28), making it an ideal metric to compare an individual's estimate against normative data (29).

Rather than comparing with a ground truth expert segmentation, the present study aimed to examine the impact of the segmentation method on the end result of a clinically motivated question, in this case, quantifying hippocampal sclerosis in patients with epilepsy. With recent progress in applying DL-based methods in medical imaging, we hypothesized that DL would provide more accurate segmentation of the hippocampi than atlas-based methods and consequently lead to improved discrimination of HS.

The experiments in this study were structured as follows: we processed T1w-MRI from healthy controls and patients with epilepsy with all four investigated methods (FS, FS-SF, FSL, and DL) using their recommended default settings. Subsequently derived measures of the hippocampal shape and volume were calculated identically from the binary segmentation of the respective method. First, we compared the impact of the segmentation method on hippocampal volumetry. Next, we identified the most important shape feature of the hippocampus using a machine-learning classifier and subsequently examined this feature for its ability to support the radiological assessment of HS. The reliabilities of the measures were assessed using repeated scans. Finally, we propose this metric as an imaging biomarker for HS along with a quantitative report to communicate the result of an individual assessment (30).

## 2. Materials and Methods

### 2.1. Dataset for Evaluation

For the evaluation, we used previously acquired data comprising healthy controls and patients with epilepsy. Included high-resolution T1-weighted MR images were acquired at the Bern University Hospital (Inselspital) on 3T scanners from Siemens (Magnetom Trio and Verio, Siemens, Erlangen, Germany) with 1 mm isotropic resolution. MR protocols were either MDEFT (31), standard 3D MP-RAGE (32), MP-RAGE according to the recommendations of the Alzheimer's Disease Neuroimaging Initiative (33), or MP-RAGE optimized for gray-white contrast (34) with sequence parameters as listed in the Supplementary Material. More than one scan was available for some subjects, resulting in a total of 126 MRIs from 105 patients and 406 MRIs from 354 healthy controls, as listed in Table 1 and Supplementary Table S1.

**Table 1:** Demographic information for the cohorts.

**Group**	**# MRI (# Subjects)**	**Mean age in years (range)**	**%Female**
Healthy Controls	406 (354)	32.3 (6.1-84.0)	57.1%
Epilepsy	126 (105)	34.7 (11.7-68.2)	52.4%
IGE/Unknown	57 (50)	32.1 (15.4-65.0)	50.9%
TLE	69 (55)	36.9 (11.7-68.2)	53.6%
HS negative	29 (24)	31.6 (12.8-57.3)	48.3%
Hippocampal Sclerosis (HS)	40 (31)	40.7 (11.7-68.2)	57.5%
Left	18 (13)	44.9 (18.5-68.2)	55.6%
Right	19 (17)	38.3 (11.7-67.9)	68.4%
Bilateral	3 (1)	31.1 (30.8-31.3)	0.0%

*Indented groups show a subset of parent line. Statistics for age and sex are calculated over the MRI samples at the time of acquisition. Corresponding information on a subject level can be found in Supplementary Table S1. IGE, idiopathic generalized epilepsy; TLE, temporal lobe epilepsy*.

The assignment of the patients' MRI to the epilepsy sub-group is based on information extracted from the radiological report of the examination (i.e., corresponds to the assessment of the neuroradiologist with the clinical and imaging information available at that time point). In particular, the initial assessment of whether HS is present was based entirely on the radiological finding. Patients without reported HS (IGE/unknown, TLE HS negative) are referred to as the “all-other-epilepsies” sub-group in the text. Where available, additional clinical and radiological information from follow-up examinations was used for a separate outlier review (cf. Section 2.5). The age of onset of the disease is known from 52 patients (with 67 MRI) with an average age of 18.4 (±14.7) years and a duration of the disease at the time of the MRI of 17.7 (±14.9) years.

### 2.2. MRI Processing

#### 2.2.1. FreeSurfer (FS)/Hippocampal Subfields (FS-SF)

The structural MRI were processed with FreeSurfer 6.0 (13) including segmentation of the hippocampal subfields (SF) using the recon_all pipeline with default parameters.

FreeSurfer extracts various morphometrics of both subcortical and cortical structures. Segmentation is performed using an anatomical atlas and Markov Random Fields (MRF) to incorporate relative spatial priors between anatomical structures and neighboring labels (12). Cortical measures are calculated on a reconstructed surface of the cortex (35). An additional module is dedicated to segment hippocampal subfields (FS-SF) using a statistical atlas built from ultra-high resolution *ex-vivo* data (14). While FS-SF internally upsamples data to a 0.3 mm resolution, our analysis was based on the results in the original 1 mm resolution to allow direct comparisons with the other methods.

#### 2.2.2. FSL-First

Segmentation of subcortical structures was generated with FSL-FIRST (15) using the fsl_anat pipeline. FIRST is available as a module distributed with FSL (36) and incorporates probability relationships between shapes and intensities using an Active Shape and Appearance Models in a Bayesian framework. For each subcortical structure, a number of *modes of variation* constrain the model, with a higher number possibly capturing more details at the cost of lower robustness. Default settings of the pipeline were used which corresponds to 30 modes for the hippocampus (15).

#### 2.2.3. DL-Based Segmentation

Deep learning-based segmentations were derived using *DL+DiReCT* (16). The tool is publicly available (https://github.com/SCAN-NRAD/DL-DiReCT) together with two models trained using a mixture of public datasets and internal data from previous studies including patients with epilepsy (as detailed in Section 2.1 of (16)) and auxiliary labels generated with FreeSurfer 6.0. Some of the MRIs in the training data were also used in the current evaluation (200 healthy controls and 60 patients with epilepsy). Therefore, to enable the reuse of these images in the current evaluation, segmentation for these images were generated using the corresponding model that has not seen these images during training.

### 2.3. Quantitative Analysis

The segmentation of the hippocampi from the four investigated methods (FS, FS-SF, FSL, and DL) were analyzed in various steps, individually per hemisphere and using the asymmetry between the hemispheres. Asymmetry indices (AI) (28) between the left (lh) and right hemisphere (rh) were calculated as follows:


(1) $$\begin{array}{r} AI\left(lh,rh\right)=\frac{lh-rh}{lh+rh} \end{array}$$

This quantity is zero for completely symmetric hippocampi and ranges between +1 and −1 otherwise.

Hippocampal volumes corrected for brain size and age were calculated for each method by fitting a linear model (lm) to the volumes of the healthy controls with the normalized (zero-mean, unit SD) co-variates *estimated total intracranial volume* (eTIV (37) from FreeSurfer) and age. In agreement with the literature (38, 39), the initially included co-variate sex was not significantly related to volumes and was subsequently removed from the model. The resulting *lm*(*vol*~*eTIV*+*age*) was then applied to *all* subjects. We then calculated effect sizes between healthy controls and left/right-sided HS for the corrected volumes.

Besides the hippocampal volumes, further metrics were extracted from the binary segmentation using *pyradiomics* (40), resulting in 14 shape features. Internally, *pyradiomics* calculated these features on a triangulated mesh generated using marching cubes (41). To identify the most important feature for further analysis, these shape features served as input to train a support vector machine (SVM) (42) to classify HS vs. all-other-epilepsies. A linear SVM (43) with default hyperparameters was trained using 5-fold cross-validation and 20 repeats. The samples were stratified by patients to ensure all MRIs from the same subject were in the same fold. Relative feature importance was aggregated across all runs to determine a feature ranking. Subsequent experiments were performed using the volume and the best-ranked shape feature.

An estimation of the discriminative power of these two metrics was determined by means of the area under the curve (AUC). Using the absolute AI to distinguish between HS (n = 37) and all-other-epilepsies (n = 86), AUC were calculated from an ROC curve (44).

**Figure 1:**
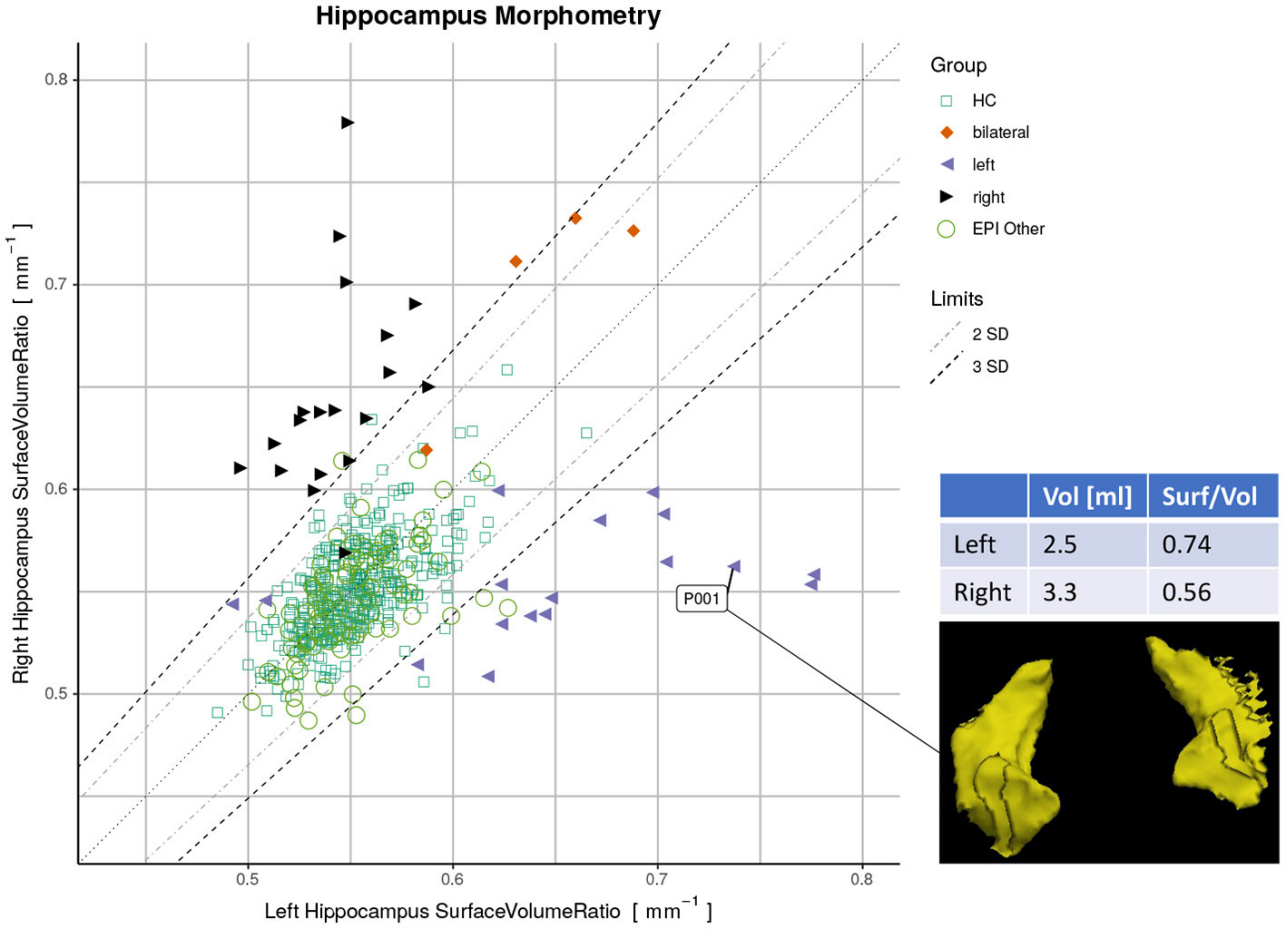
Proposed reporting for the suggested hippocampal sclerosis (HS) biomarker by plotting the surface-to-volume ratio of both hippocampi in one datapoint. Healthy controls (HC) serve as normative data with left and right-sided HS predominantly appearing outside the limits. The highlighted case with an atrophic hippocampus on the lateral side is of a left-sided HS (appearing on the right side of the rendering in radiological orientation).

Finally, we used a quantitative report as outlined in Figure 1 to display the ratios of both hippocampi simultaneously. Exact symmetries would appear along the diagonal line. Standard deviations (SD) of the asymmetry indices (AI) in the healthy controls (n=406) were calculated to demarcate limits of two and three SD from the expected norm. Based on these limits, accuracy metrics (sensitivity, specificity, and F1-score) were determined for classifying unilateral HS vs. all-other-epilepsies.

Statistical analyses were performed using *R* with the *stats* package version 3.6.2 (45). Effect sizes were reported using Cohen's *d* (46). A significance level of α = 0.05 was set.

### 2.4. Robustness

To assess the robustness of the methods, we have used the same-day repeated scans in the dataset and determined a reproducibility error. For each metric *m*, we calculated the mean absolute percentage error (MAPE) as follows:


(2) $$\begin{array}{r} MAPE=\frac{100}{N}\overset{N}{\underset{i=1}{\sum }}\left(\frac{1}{n\left(i\right)}\overset{n\left(i\right)}{\underset{t=1}{\sum }}\frac{\left|m_{\left(i,t\right)}-\mu_{\left(i\right)}\right|}{\mu_{\left(i\right)}}\right) \end{array}$$

where *N* is the number of sessions with re-scans, *n*(*i*) the number of re-scans in the session *i* for a subject, *m*_(*i, t*)_ the measurement at timepoint *t*, and $\mu_{\left(i\right)}=\frac{1}{n\left(i\right)}\overset{n\left(i\right)}{\underset{t=1}{\sum }}m_{\left(i,t\right)}$ the within-session mean. A session comprises the scans acquired on the same day for the patients and all scans within 1 year for the healthy controls, resulting in 41 sessions. Statistical significance of the differences between the four methods was determined using paired *t*-tests.

Additionally, we have calculated intraclass correlation coefficients (ICC) with the first two MRIs from every session. The random effects of repeated acquisitions are reflected in a *two-way random-effect model with an absolute agreement*, also known as *ICC*(2, 1) (47, 48), implemented in the R-package *irr* (49).

### 2.5. Outlier Review

As outlined above (Section 2.1), the patients' MRIs were initially assigned to epilepsy sub-groups entirely based on information from the radiological report of the corresponding image. Therefore, we performed an additional sensitivity analysis. An experienced radiologist (co-author PR) reviewed all ‘*wrongly'* classified cases, i.e., putative false negatives (HS appearing inside the limits of 3 SD relative to healthy controls) and false positives (all-other-epilepsies appearing outside the limits). For the review, all available clinical information was taken into account including patient history, follow-up assessments by epileptologists, further diagnostics like EEG, additional MRI examinations, and all medical reports. Results after correcting the assigned sub-groups are reported separately.

### 2.6. Comparison to Other DL-Based Methods and Manual Tracing

In a supplementary subanalysis, we compared the results from DL+DiReCT to two other DL based segmentation methods: the whole-brain neuroanatomy segmentation method FastSurfer (19)^1^ and HippoDeep (21)^2^, which specifically segments the hippocampi only.

Additionally, we report surface-to-volume ratios derived from the manual tracing of the hippocampi by experts as provided in the *OASIS TRT-20* dataset (50, 51) of twenty healthy individuals.

## 3. Results

### 3.1. Hippocampal Volumetry

**Figure 2:**
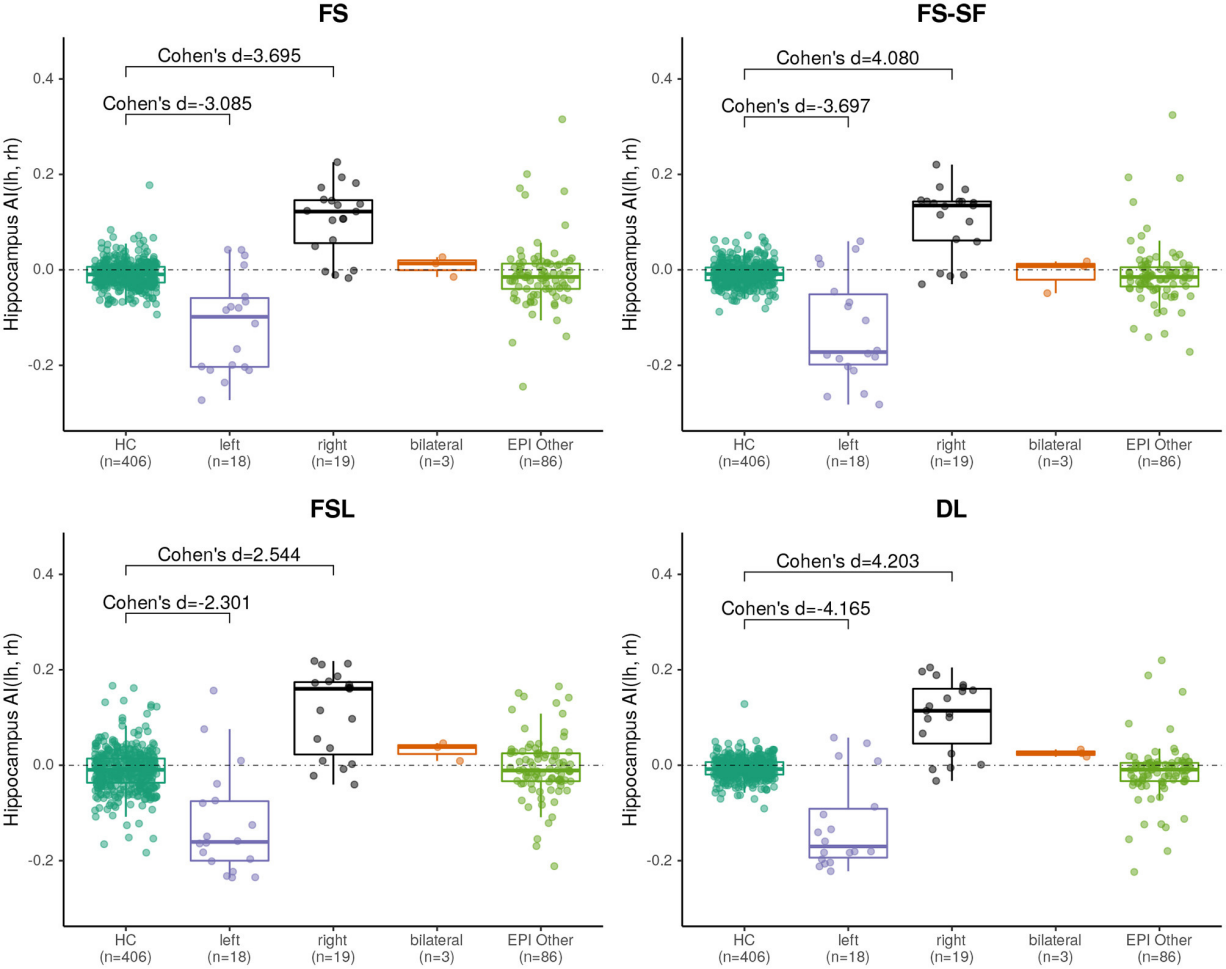
Boxplot of the asymmetry indices (AI) of hippocampus volumes derived from the four segmentation methods. Effect sizes indicate difference between healthy controls (HC), and left/right-sided HS.

Effect sizes of hippocamal volumes (after correction for brain size and age) between healthy controls and HS were larger for DL (left hippocampus = −2.968, right= −1.904) than for FS (left= −2.462, right= −1.624), FS-SF (left= −2.376, right= −1.661), and FSL (left= −2.818, right= −1.826) with detailed distributions reported in Supplementary Figures S1,S2. The asymmetry index (AI) of the volumes (uncorrected as the contralateral side serves as internal reference for every MRI) were generally more sensitive, again with the effect sizes for DL being larger (HS left= −4.165, right = 4.203) than for FS (left= −3.085, right= 3.695), FS-SF (left= −3.697, right = 4.080), and FSL (left= −2.301, right = 2.544) as shown in Figure 2. In healthy controls, a statistically significant (*p* <10^−5^) negative mean AI was observed from all four methods (FS= −0.010, FS-SF= −0.008, FSL= −0.011, and DL = −0.007), indicating a slightly larger right hippocampal volume.

**Figure 3:**
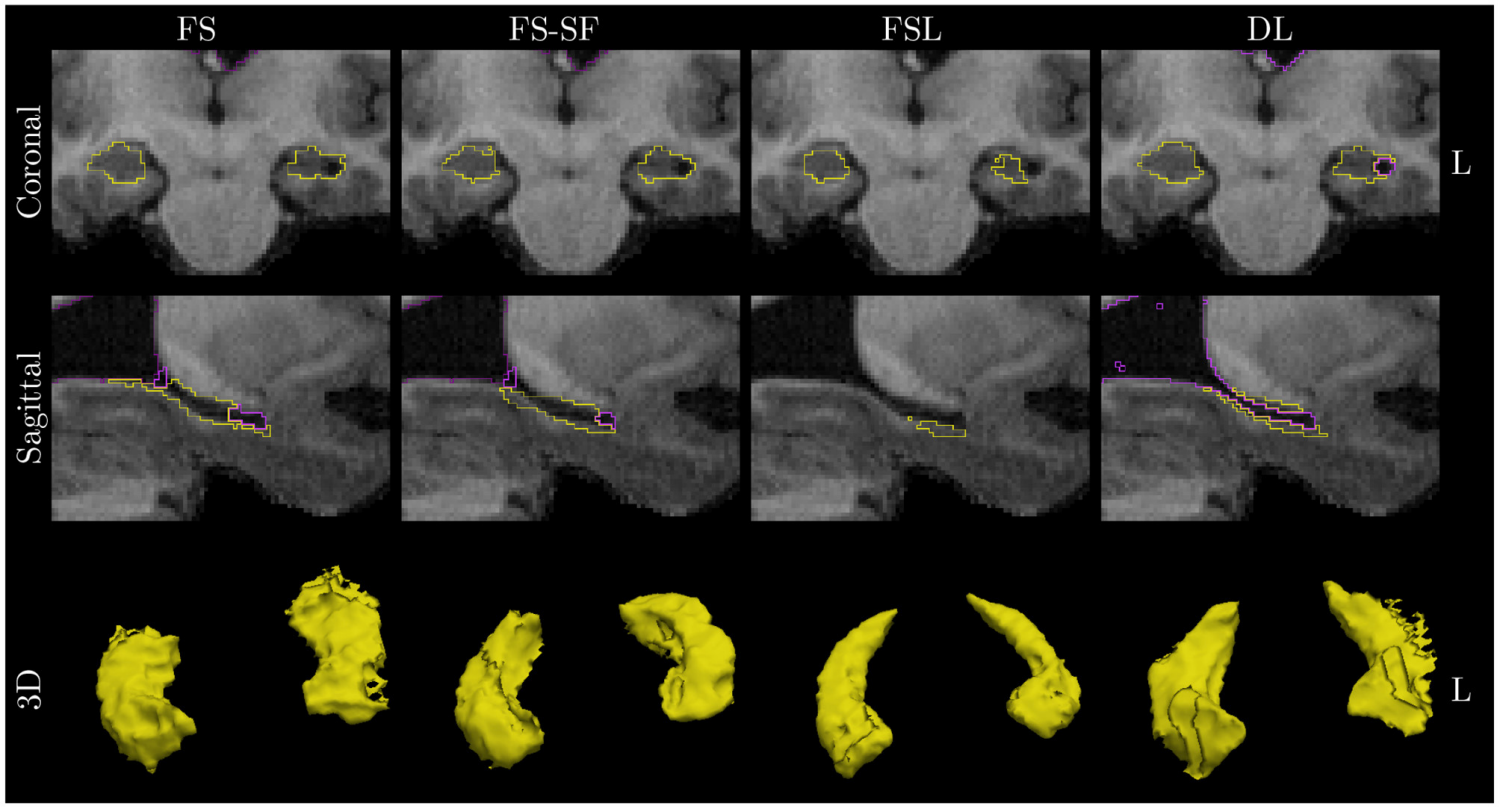
Qualitative example of a case with left HS. Images are in radiological orientation, i.e., the left (L) hemisphere appears on the right side of the image. Boundaries of the segmentation are outlined for the hippocampi (yellow) and ventricles/CSF (purple). Coronal view of the hippocampal body and sagittally of the left hippocampus. While FS correctly identified fluid-filled cavities at the tail and head of the hippocampus, this was only fully captured by deep learning (DL) along the entire body of the hippocampus. The example corresponds to the case highlighted in Figure 1.

A qualitative example of a patient with left-sided HS is shown in Figure 3 with automated segmentation of the hippocampi and ventricles outlined (as shown in Supplementary Figure S3 for additional examples). Qualitatively inspecting the results, mis-segmentation by the atlas-based methods was most frequently observed on the lateral side of the body of atrophic hippocampi.

### 3.2. Surface-to-Volume Ratio

The surface-to-volume ratio was identified by the SVM as the most important shape feature from the DL-based segmentation (Supplementary Figure S5).

**Figure 4:**
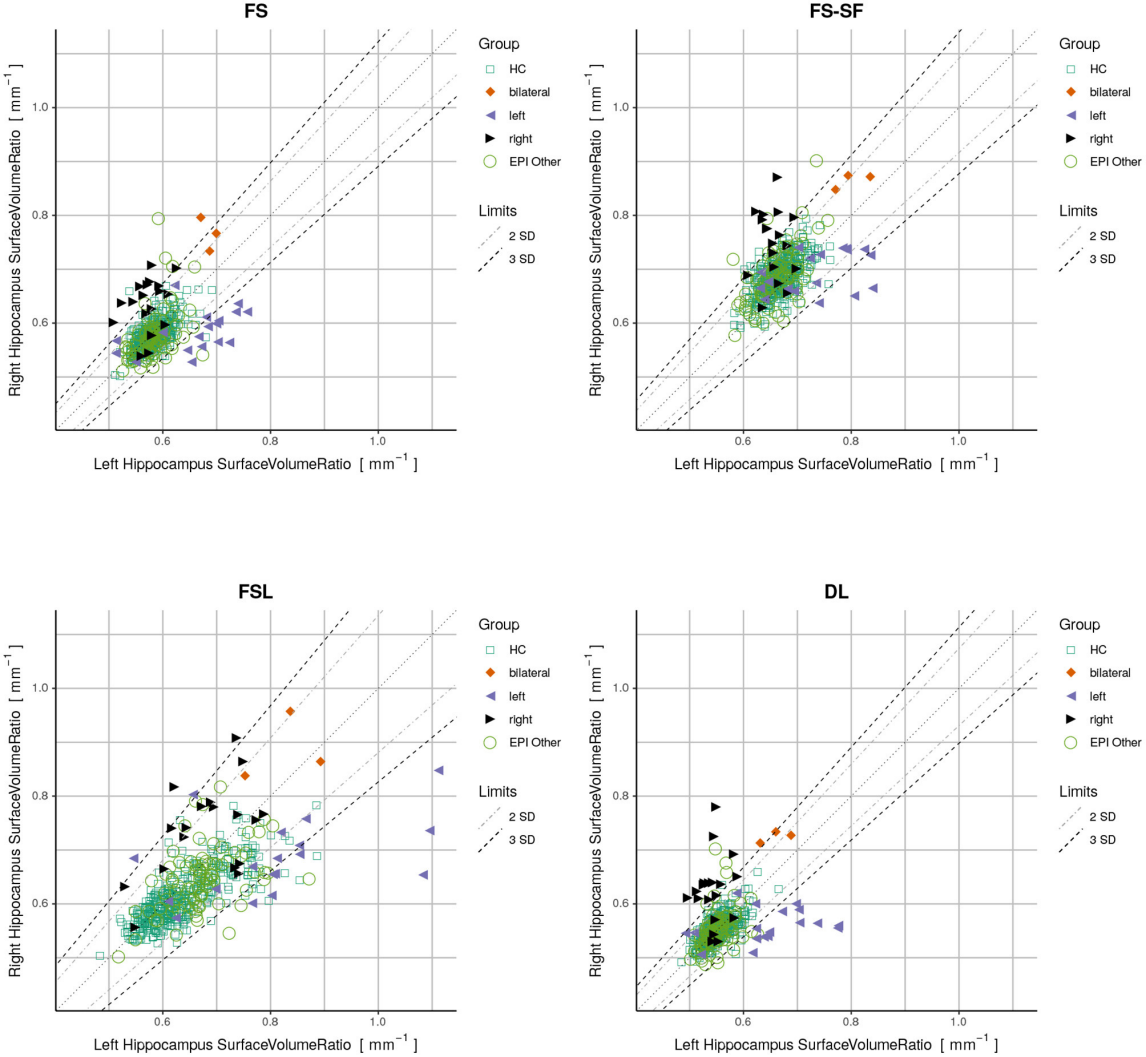
Plots displaying surface-to-volume ratio of left (x-axis) and right (y-axis) hippocampi derived from the four segmentation methods. Healthy controls (HC), hippocampal sclerosis (bilateral/left/right), and all-other-epilepsies (EPI Other) are color-coded. Limits showing two and three standard deviations (SD) calculated on the HC.

By plotting the left vs. the right surface-to-volume ratios in Figure 4, we can observe the healthy controls and all-other-epilepsies clustering along the diagonal while left-sided HS appear on the lower right triangle, right-sided HS on the upper left, and the bilateral cases toward the upper right corner, indicating that in contrast to the volume, the surface-to-volume ratio **increases** in the presence of HS.

**Figure 5:**
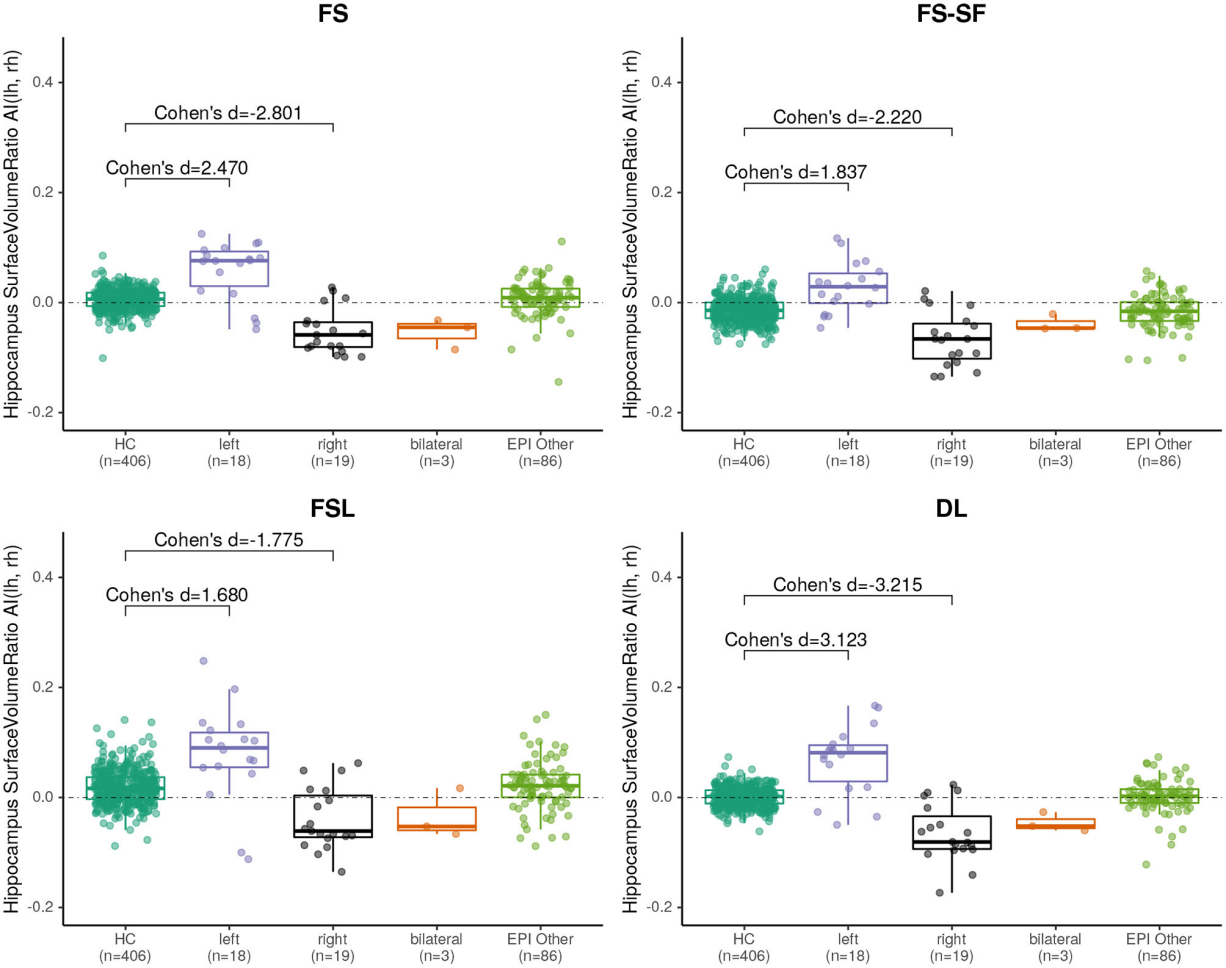
Boxplot of the asymmetry indices (AI) of hippocampus surface-to-volume ratios derived from the four segmentation methods. Effect sizes indicate difference between healthy controls (HC), and left/right-sided hippocampal sclerosis.

We have observed a symmetric ratio in the healthy controls only from the DL-based segmentations (*p* = 0.24, two-sided t-test for asymmetry), whereas the other three methods had either a significantly (*p* <10^−8^) positive (FS, FSL) or negative (FS-SF) ratio. Separation of point clouds (Figure 4) and effect sizes between healthy controls and HS, as reported in Figure 5, were generally larger for DL than the other three methods.

**Figure 6:**
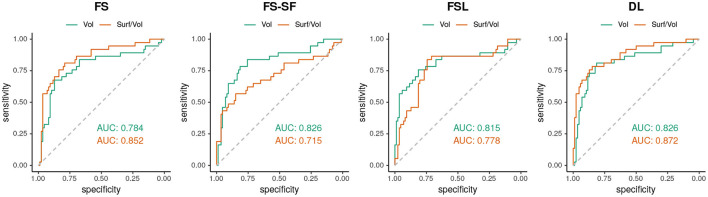
ROC-curves using the absolute asymmetry index (AI) to separate between HS and all-other-epilepsies.

When classifying MRIs using 3 SD of AI on the healthy controls as a threshold, DL reached the highest accuracy in terms of F1-score (Supplementary Table S2) both for the volume (*F*1 = 70.0) and for the surface-to-volume ratio (*F*1 = 71.2) which is consistent with the highest AUC observed (Figure 6) for the DL-based segmentation.

### 3.3. Robustness

A comparison of the robustness evaluation from the re-scan sessions (*n* = 41) is listed in Table 2. The surface-to-volume ratio derived by the DL-based segmentation was statistically significantly more robust (lower MAPE) than the other three methods (Supplementary Figure S8). For the volume, only FSL was comparably robust to DL. For FS and DL, reproducibility by means of ICC was generally higher for the surface-to-volume ratio than for the volume.

**Table 2:** Robustness in terms of mean absolute percentage error (MAPE) and intraclass correlation coefficient (ICC).

	**MAPE**				**ICC(2,1)**			
	**FS**	**FS-SF**	**FSL**	**DL**	**FS**	**FS-SF**	**FSL**	**DL**
Left hippocampus volume	2.957%	3.870%	**1.749%**	1.921%	0.867	0.782	**0.931**	0.918
Right hippocampus volume	3.435%	4.371%	2.802%	**2.101%**	0.796	0.696	0.844	**0.882**
Left hippocampus surface/volume	1.147%	1.893%	2.765%	**0.663%**	0.890	0.766	0.643	**0.955**
Right hippocampus surface/volume	1.025%	1.905%	2.554%	**0.738%**	0.927	0.722	0.750	**0.941**

*Bold numbers highlight the lowest MAPE and highest ICC in every row*.

### 3.4. Outlier Review

When classifying MRIs like described above with the DL-based method, 21 cases (16.7% of all MRI, from 17 patients) were putative false negatives or false positives in relation to the initial radiological assessment serving as ground truth. MRIs stemming from the same patient (7 MRIs from three patients, as shown in Supplementary Table S3) appeared in close vicinity in the quantitative report (Supplementary Figure S6). After reviewing all medical records by an expert, this diagnosis was confirmed in 8 of these 17 patients. However, the other nine patients were classified differently after considering all follow-up clinical information. These outliers are listed in Supplementary Table S3 and highlighted in Supplementary Figure S6 and the resulting plot with the corrections is shown in (Figure 1). AUC for the classification with the adjusted classes was accordingly higher (see Supplementary Figure S7), while only minimally disturbing the order of the effect sizes.

### 3.5. Comparison to Other DL-Based Methods and Manual Tracing

The supplementary comparison of results from three different DL-based methods can be found in Supplementary Section 6. In the surface-to-volume plots (Supplementary Figure S9), the healthy individuals from the OASIS dataset cluster around the healthy controls for the DL-based methods whereas the ratios are significantly higher from the manual tracing.

## 4. Discussion

In this study, we compared a DL-based neuroanatomy segmentation method to three established and commonly used atlas-based methods. Specifically focusing on the hippocampus, we assessed how the quality of the segmentation impacts metrics used to quantify HS in patients with epilepsy. Shape features derived from the segmentation were examined for their discriminative power and reliability.

FreeSurfer has been reported to be more accurate than FSL-FIRST compared to manual segmentation of the hippocampus (9, 52, 53), consistent with our observations of higher agreement among the other three methods (Supplementary Figures S1,S2). Automated methods have shown reduced accuracy in pathological cases (9, 54, 55) as well as systematic bias in younger age groups (56) for which cohort-specific atlases have been recommended (57). However, hippocampal atrophy of patients with TLE might be accompanied by atypical shape and positioning of the hippocampus (58) which would require choosing from disease-specific templates (59–61). In this study, we have observed DL generating more plausible segmentation in pathological cases, suggesting the superiority of learning-based methods possibly due to a large number of variable training samples.

We observed that mis-segmentation of the atlas-based methods were often on the lateral side along the body of atrophic hippocampi (cf. Figure 3). We suspect this is due to the lower prior probability of fluid-filled cavities in this region as it was observed to a lesser extent toward the tail (closer to the lateral ventricle) and head (inferior horn of lateral ventricle). In particular, for FSL-FIRST, this might be caused by the relatively low number of modes used in the default settings (9, 15).

All methods revealed a negative asymmetry index (AI) of hippocampal volumes in the healthy controls, indicating a slightly larger right hippocampus, which is a well-documented observation in the literature, reported for FreeSurfer on a very large cohort (mean AI of -0.007, identical to the result of our method for a 40-fold smaller sample of controls) (28), using manual tracing (62), and in a meta-analysis (63). This is probably also the cause for the observed larger effect sizes of HS-right (cf. Figure 4).

An often-cited limitation of supervised learning-based methods is the sparsity of (manually) annotated training data. Our results suggest that such a model can be trained entirely with weak labels, which can be generated automatically in large quantities using established tools like FreeSurfer (16, 64). Interestingly, the predictions of such a trained model seem to be at least as robust and potentially more sensitive than the method used to generate the training data.

The sensitivity of detecting HS is significantly lower with standard MRI than with epilepsy specific protocols, particularly if performed by less experienced radiologists (6). Our DL-based segmentation runs in about two minutes, including radiomics, substantially faster than several hours of processing time for FreeSurfer without surface reconstruction. The almost immediate availability is an advantage for future applications in clinical routine. The fast processing time would even allow a preliminary analysis during scanning of the patient and potentially suggesting further hippocampus-specific protocols in case of (semi-automatic) detection of hippocampal abnormalities while the patient is still in the scanner.

Hippocampal volumetry is the most common method to quantify HS. However, volume loss can be subtle and does not reflect other traits of a degenerating hippocampus. For example, a frayed CA1 region might also broadly impact the surface area, making the ratio of the two quantities a potentially more specific measure. The ratio might also be helpful for bilateral HS cases where the AI of surface-to-volume ratio seems to be qualitatively more discriminative (cf. Figure 4) than from volume (cf. Supplementary Figure S4). Overall the ratio was slightly more discriminative (cf. Figure 6) and showed a higher reproducibility across repeated scans (cf. ICC in Table 2) than the volume alone.

Radiomics features have been suggested before for detecting HS (65, 66), often by combining a plethora of different features, which makes the interpretation difficult. In our experiments, the surface-to-volume ratio was identified by an SVM classifier as the most important metric out of 14 shape features. Moreover, it is a feature that is immediately understandable by non-technical personnel as it is a biologically plausible metric for hippocampal sclerosis. Such an easy-to-interpret quantitative imaging biomarker for HS could potentially increase the acceptance and facilitate communication of findings with clinicians. We proposed to report such a biomarker by plotting the left against the right measures along with normative data (cf. Figure 1) which has the advantage to make asymmetries visible (deviations from the diagonal) as well as show the absolute values in a single data point.

### 4.1. Limitations

As we consider manual segmentation not a viable option for a potential future clinical application, the aim of the study was designed to compare an efficient DL-based method against commonly used atlas-based methods without comparison against a manually derived ground truth. To account for this limitation, we demonstrated the influence of manual labels on the proposed surface-to-volume ratio with data from *Mindboggle* (51), a frequently used publicly available dataset with manually annotated neuroanatomy labels. Although manual tracings are generally looking good on the coronal view, we confirm the earlier observation of disturbing *staircaise* effects by Coupé et al. (67) in the axial and sagittal view of manually traced hippocampi (cf. Supplementary Figures S10,S11). This demonstrates the difficulty of manual tracing and the challenge for humans to label 3D structures in 2D views. A remedy would require correcting tracings from all three directions iteratively until complete consistency, which is difficult for the hippocampus as tracing protocols for the hippocampus are predominantly defined in the coronal direction (68). While these slice inconsistencies probably have a less pronounced effect on the calculated volumes due to averaging effects, the surface area is particularly prone to such artifacts. Consequently, manual tracing is not an option for this type of shape analysis.

While the primary analysis aimed to compare DL vs. atlas-based methods and not necessarily find the best DL-based algorithm, we replicated key figures and metrics with two other popular deep learning methods in a supplementary analysis. Overall, the DL-based methods yielded comparable results, outperforming atlas-based methods.

All segmentation methods were used with their default settings (without hyperparameter tuning on the dataset), recognizing this might have caused FSL-FIRST to underperform in this comparison due to the low *number of modes*. Results from all methods were used as is without manual corrections. The dataset contained T1w images with minor variations in the MR protocol which might influence the segmentations.

We have not performed an in-depth shape analysis of the segmented hippocampi but rather used simple metrics to demonstrate that an improved segmentation leads to better discrimination of abnormal hippocampi. We speculate that advanced shape analysis techniques (55, 69, 70) would benefit from the improved DL-based segmentation.

A varying amount of information was available for assigning the patients' MRIs to the epilepsy sub-groups. Therefore, we have deliberately used the initial radiological diagnosis as ground truth for the primary analysis. To account for uncertainty in the diagnosis, we performed an outlier review by an imaging expert and reported these results separately. Some cases changed diagnosis after reviewing all follow-up clinical information, confirming the challenge of diagnosis HS from MRI.

### 4.2. Outlook

In visual assessments of suspected HS, the T2w image contains important additional information for the reader. It remains to be investigated whether supplying the corresponding T2w as an additional input to the model can help to further improve the segmentation. Quantitative methods analyzing T2 or FLAIR intensities in a region of interest (30, 71) might also benefit from an improved segmentation of the hippocampi.

The data in this evaluation were predominantly of patients with longer disease duration. We will subsequently apply the method in a multi-center prospective study of first-seizure patients (72) to assess its utility in early-onset epilepsies. Providing the proposed metrics together with the MRI to neuroradiologists could be useful in the clinical routine.

## 5. Conclusions

Our findings suggest that deep learning-based neuroanatomy segmentations are more sensitive and robust than atlas-based methods to support radiological assessments of HS in MRI of patients with epilepsy. Beyond volumetry of the hippocampus, the surface-to-volume ratio further increases the discriminative power to dichotomize HS from other epilepsies while being a more robust metric. It could serve as a potential quantitative imaging biomarker of interest for HS.

## Data Availability Statement

The datasets presented in this article are not readily available because the experiments were performed with data from patients and healthy controls of the Bern University Hospital. All study participants signed informed consent for the use of their data for research. However, this did not include permission to make the raw data publicly available.

## Ethics Statement

The studies involving human participants were reviewed and approved by Kantonale Ethikkommission Bern (protocol 2017-00697). Written informed consent to participate in this study was provided by the participants' legal guardian/next of kin.

## Author Contributions

CR, MiR, and PR: conceive the project idea. MiR, CR, and PR: design of experiments. MiR, RM, and MaR: deep-learning network architecture. MiR: perform experiments, data analysis, and manuscript drafting. PR: radiological assessment of cases. CR, PR, and RW: manuscript revision. All authors reviewed and approved the final version of the manuscript.

## Funding

This work was supported by the Swiss National Science Foundation under grant number CRSII5_180365 (The Swiss-First Study).

## Conflict of Interest

The authors declare that the research was conducted in the absence of any commercial or financial relationships that could be construed as a potential conflict of interest.

## Publisher's Note

All claims expressed in this article are solely those of the authors and do not necessarily represent those of their affiliated organizations, or those of the publisher, the editors and the reviewers. Any product that may be evaluated in this article, or claim that may be made by its manufacturer, is not guaranteed or endorsed by the publisher.

## Footnotes

^1^ https://github.com/Deep-MI/FastSurfer

^2^ https://github.com/bthyreau/hippodeep_pytorch
