# Supplementary material for: A Quantitative Imaging Biomarker Supporting Radiological Assessment of Hippocampal Sclerosis Derived From Deep Learning-Based Segmentation of T1w-MRI
Source: Front Neurol. 2022 Feb 18;13:812432. doi: 10.3389/fneur.2022.812432 (PMC8894898; doi:10.3389/fneur.2022.812432)
Supplement: Supplementary file 1 [file Data_Sheet_1.pdf]

Supplementary Materials for
A Quantitative Imaging Biomarker supporting Radiological Assessment of Hippocampal Sclerosis derived from Deep Learning-based Segmentation of T1w-MRI

Contents

1 Demographic Information 2

2 Hippocampus Segmentation 3

3 Feature Importance of SVM Classifier 7

4 Outlier Review 8

5 Robustness 10

6 Comparison to other DL-based Methods and Manual Tracing 11

1 Demographic Information

Group	# Subjects	Mean age in years (range)	%Female
Healthy Controls	354	32.5 (6.1-84.0)	53.7%
Epilepsy	105	35.3 (11.7-68.2)	49.5%
IGE/Unknown	50	32.9 (15.4-65.0)	46.0%
TLE	55	37.5 (11.7-68.2)	52.7%
HS negative	24	32.0 (12.8-57.3)	50.0%
Hippocampal Sclerosis (HS)	31	41.7 (11.7-68.2)	54.8%
left	13	45.0 (18.5-68.2)	46.2%
right	17	39.9 (11.7-67.9)	64.7%
bilateral	1	31.1 (31.1-31.1)	0.0%

Table S1: Demographic information for the cohorts based on the unique subjects (opposed to information on the level of the MRI in the main text). Indented groups show a subset of parent line. IGE: idiopathic generalized epilepsy, TLE: temporal lobe epilepsy.

2 Hippocampus Segmentation

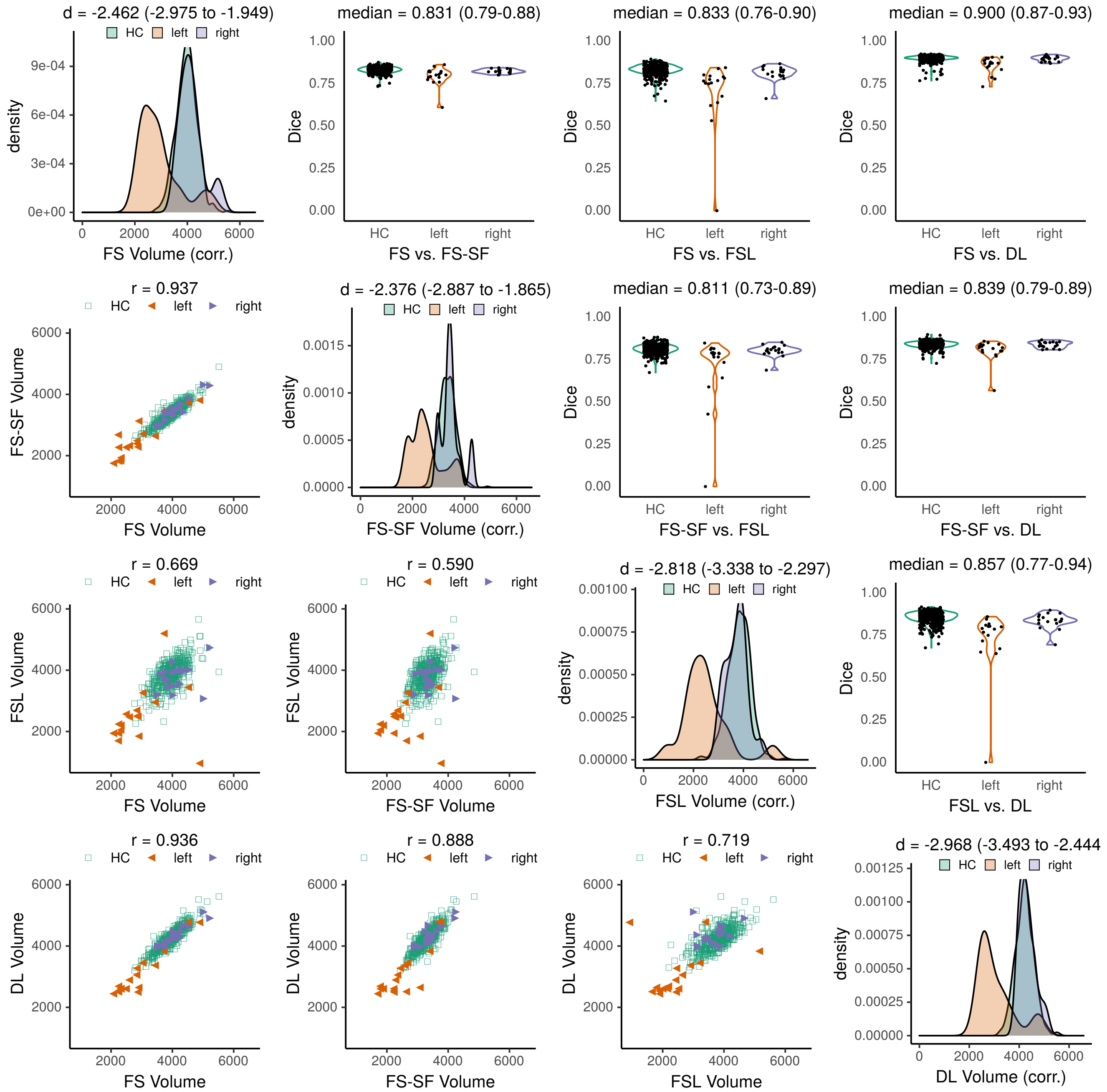

Figure S1: Plots organized as matrix with various metrics for the **left** hippocampus. For better readability, only healthy controls (HC) and left/right HS sub-groups are shown (without bilateral HS and all-other-epilepsies). On the diagonal, density distribution of hippocampal volumes (corrected for brain size and age) are shown with effect sizes (Cohen's d) between healthy controls and left/right HS. In the lower triangle of the matrix, correlations of hippocampal volumes between all the methods are shown. The upper triangle shows spatial overlap between the segmentation by means of Dice coefficient as boxplot and median ($1.5\times$ interquartile range in the title).

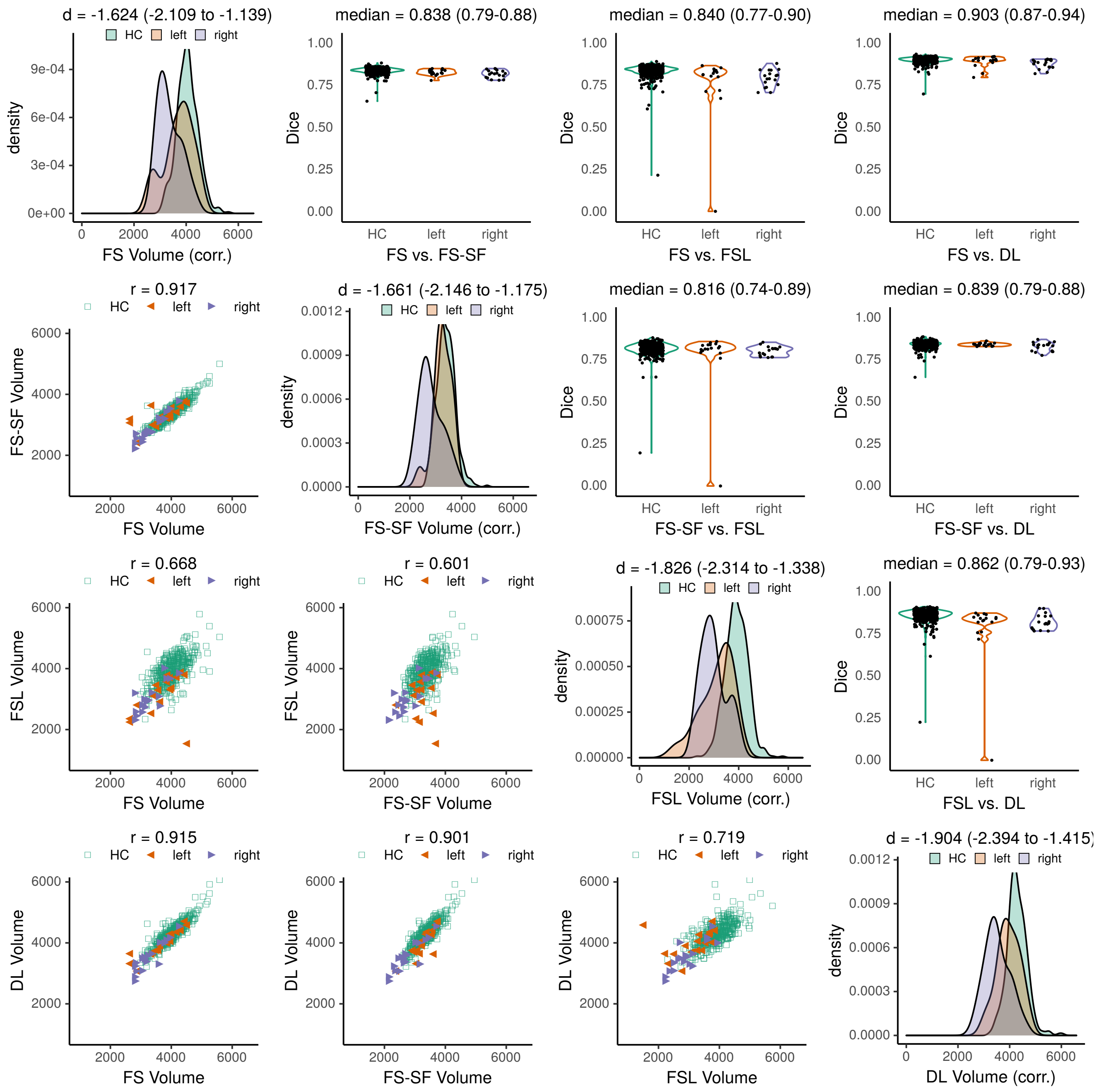

Figure S2: Plots organized as matrix with various metrics for the **right** hippocampus. For better readability, only healthy controls (HC) and left/right HS sub-groups are shown (without bilateral HS and all-other-epilepsies). On the diagonal, density distribution of hippocampal volumes (corrected for brain size and age) are shown with effect sizes (Cohen's *d*) between healthy controls and left/right HS. In the lower triangle of the matrix, correlations of hippocampal volumes between all the methods are shown. The upper triangle shows spatial overlap between the segmentation by means of Dice coefficient as boxplot and median (1.5× interquartile range in the title).

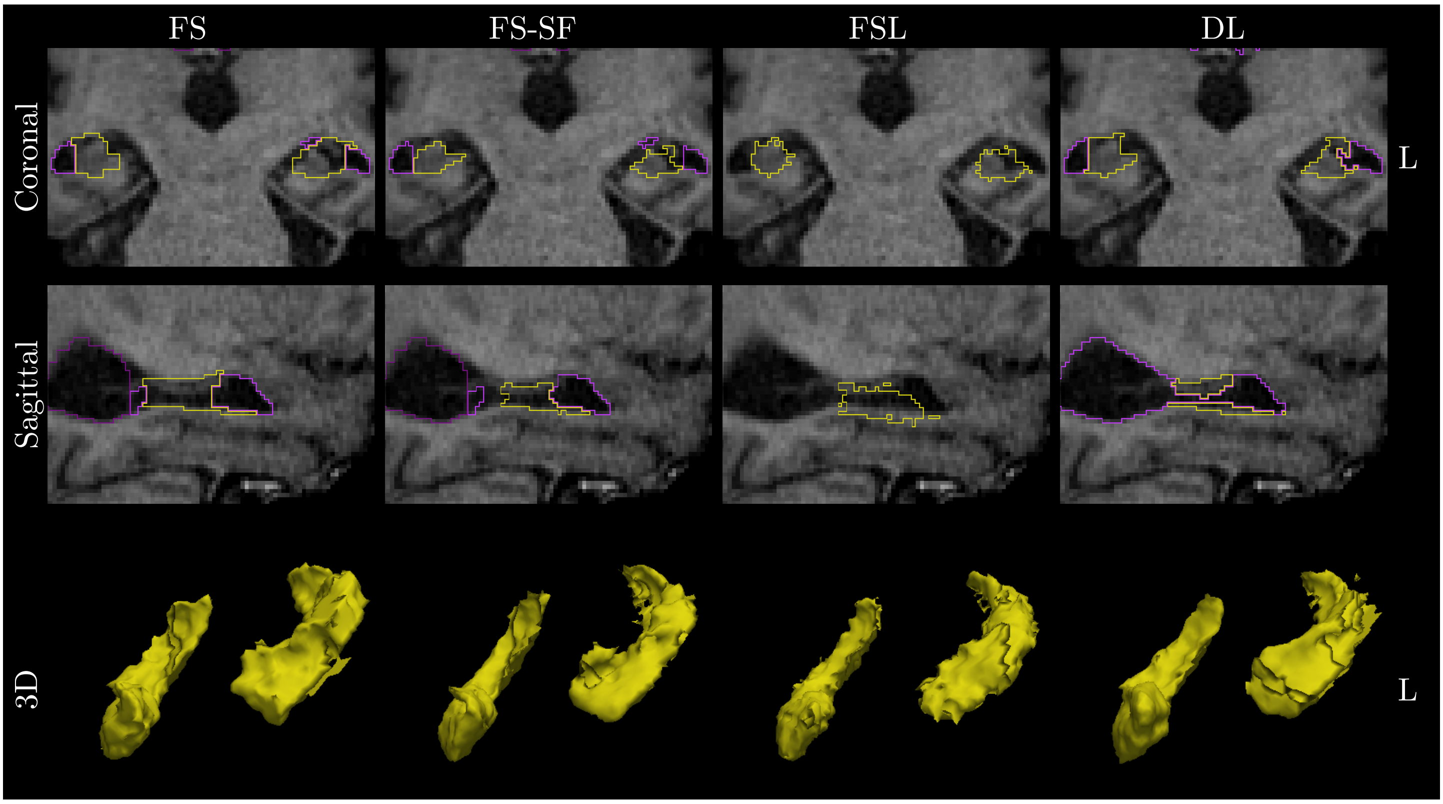

Figure S3: Qualitative example for the segmentation of case P017 with left hippocampal sclerosis. Images are in radiological orientation, i.e. the left (L) hemisphere appears on the right side of the image. Boundaries of the segmentation are outlined for the hippocampi (yellow) and ventricles/CSF (purple). Coronal view of the hippocampal body and sagittally of the atrophic left hippocampus.

	FS			FS-SF			FSL			DL			FastSurfer			HippoDeep		
	Sens.	Spec.	F1	Sens.	Spec.	F1	Sens.	Spec.	F1	Sens.	Spec.	F1	Sens.	Spec.	F1	Sens.	Spec.	F1
AI Vol.	62.2	87.2	64.8	67.6	83.7	65.8	59.5	**91.9**	66.7	**75.7**	82.6	70.0	70.3	82.6	66.7	73.0	88.4	**73.0**
AI Surf./Vol.	59.5	93.0	67.7	40.5	**96.5**	54.5	32.4	93.0	43.6	**70.3**	88.4	71.2	67.6	93.0	**73.5**	**70.3**	90.7	73.2

Table S2: Accuracies of classifying the epilepsy cases with regard to the 3 SD calculated on the asymmetries of the healthy controls (cf. Figure S4). Bold numbers highlight the highest sensitivity/specificity/F1 score per row.

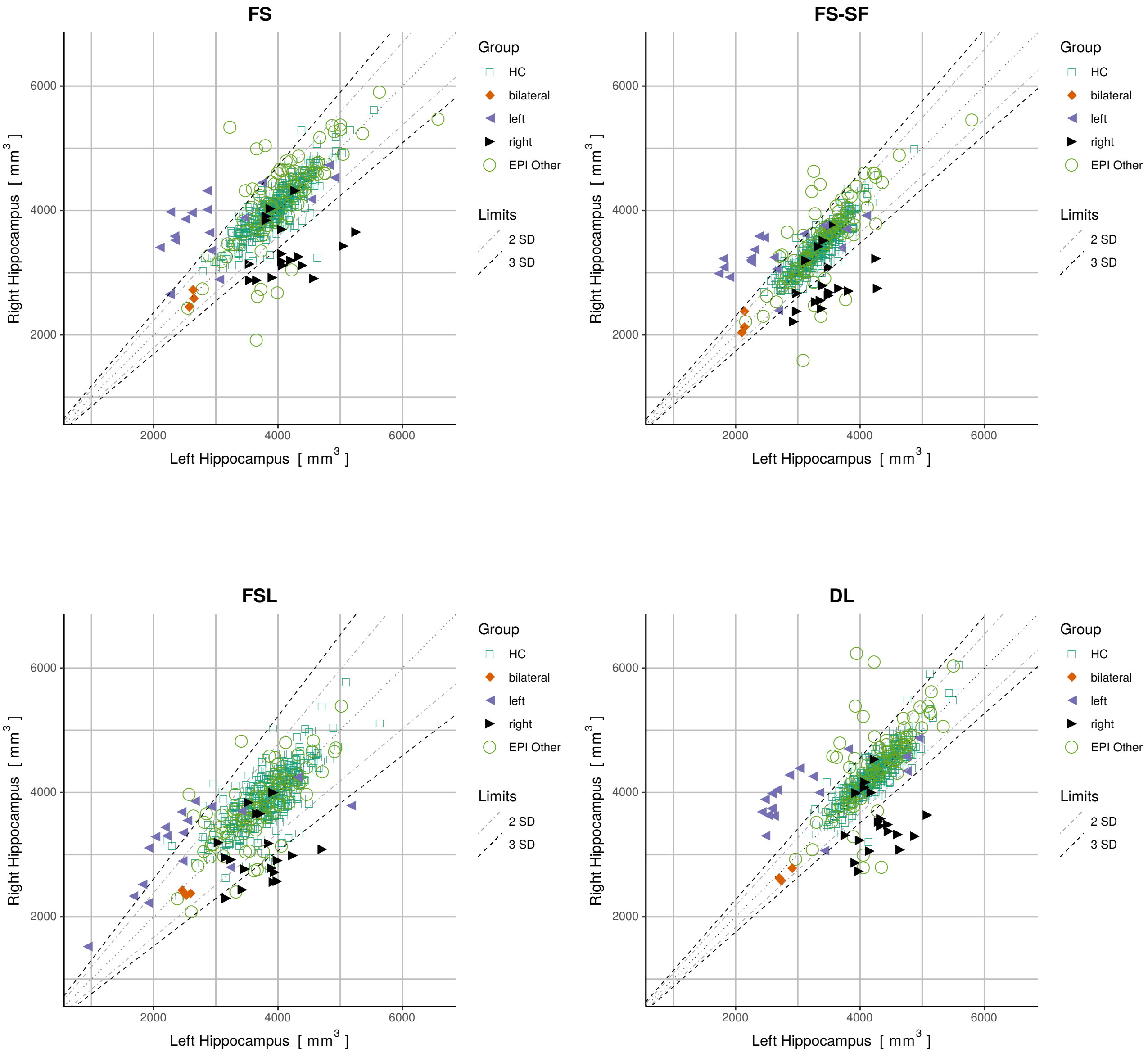

Figure S4: Plots displaying the **volume** of the left (x-axis) and right (y-axis) hippocampi derived from the four segmentation methods. Healthy controls (HC), hippocampal sclerosis (bilateral/left/right), and all-other-epilepsies are color-coded. Limits showing two and three standard deviations (SD) calculated on the HC. As opposed to the surface-vol-ratio in the main text, the bilateral cases appear shifted towards origin of the plot.

3 Feature Importance of SVM Classifier

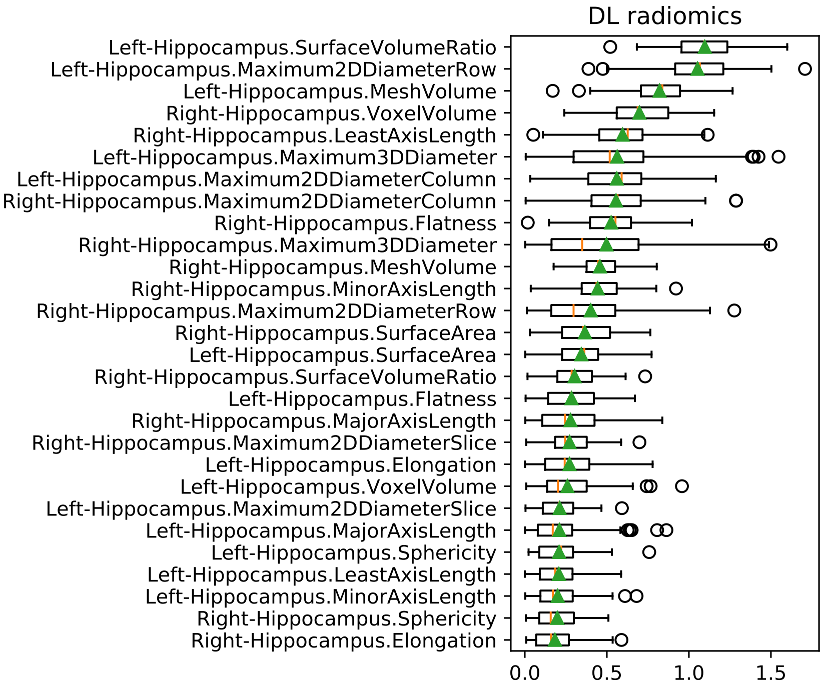

Figure S5: Relative feature importance from the SVM classifying HS vs. non-HS. Aggregated feature importance from a repeated five-fold cross-validation is shown as boxplot.

4 Outlier Review

MRI ID	Initial Diagnosis	DL Classified as	Revised Diagnosis	Clinical and Diagnostic Summary
P012,P104	HS left	non-HS	-	Pharmaco-resistent TLE (left HS), sAHE left, Outcome Engel Ic.
P016	HS left	non-HS	-	Radiological suspicion MTLE left, EEG suggesting an epileptogenic focus in the right temporal lobe.
P018	HS left	non-HS	HS bilateral	Pharmaco-resistant structural epilepsy with complex focal seizures. Unclear findings in the left hippocampus and amygdala (differential diagnosis of post-inflammatory alterations, focal cortical dysplasia, low-grade glioma with secondary secondary hippocampal pathology), discrete signs of HS also on the right side.
P025	HS right	non-HS	-	Pharmaco-resistent structural MTLE, histologically confirmed HS right, Outcome Engel Ia.
P036	HS left	non-HS	non-HS	Symptomatic epilepsy with complex focal and secondary generalized seizures. Suspicion of DNET in left temporal lobe, surgery (no histpathological report available), Outcome Engel Ia.
P040,P041,P103	HS right	non-HS	non-HS	Pharmaco-resistant structural epilepsy, diffuse astrocytoma WHO grade II, cortectomy temporoparietal right, Outcome ILAE 4.
P205	FLE	HS left	-	no further information.
P227	HS right	non-HS	non-Epi	epilepsy not confirmed.
P228	TLE non-lesional	HS right	HS right	Pharmaco-resistent TLE (right HS), sAHE right, Outcome Engel Ia.
P232	TLE left	HS left	HS left	Left temporopolar substance defect after ICB from AVM (AVM extirpation), left HS, left anterior temporal lobe resection with AHE, Outcome Engel Ia.
P264	FLE right	HS right	-	Structural epilepsy after TBI with right frontotemporal lobe contusion, surgical lobectomy frontotemporal, Outcome Engel Ia.
P266	FCD mesiotemporal	HS left	-	TLE right, suspicion of FCD or DNET, no surgery.
P275,P276	FLE right	HS left	-	Pharmaco-resistant non-lesional epilepsy, 1st surgery amygdalectomy and 1/3 lobectomy (anterior temporal) right, 2nd surgery: completing amygdalectomy, hippocampectomy, and anterior temporal lobectomy.
P280	MTLE non-lesional	HS right	HS right	Pharmaco-resistent TLE (right mesiotemporal sclerosis), sAHE, Outcome ILAE 1.
P281	Unknown	HS right	HS right	Structural epilepsy after TBI, right HS, no surgery
P284	TLE right	HS right	HS right	Structural focal epilepsy, tuberculosis meningitis, right HS (suspected on MRI, correlating EEG findings), sAHE, Outcome Engel Ia.
P285	HS right	non-HS	-	Structural epilepsy with right mesiotemporal sclerosis (MRI), probably post-inflammatory, seizure classification: dyscognitive and secondary bilateral convulsive seizures, no surgery.

Table S3: Result of the manual review of all cases identified as outlier in the surface-to-volume plot of DL highlighted in Figure S6. Multiple IDs are listed where several MRI are available for an individual. Clinical and diagnostic summary using most recent available data. (M)TLE: (mesial) temporal lobe epilepsy. (s)AHE: (selective) amygdala hippocampectomy. TBI: traumatic brain injury. Outcome: post-surgical seizure control (Engel or ILAE).

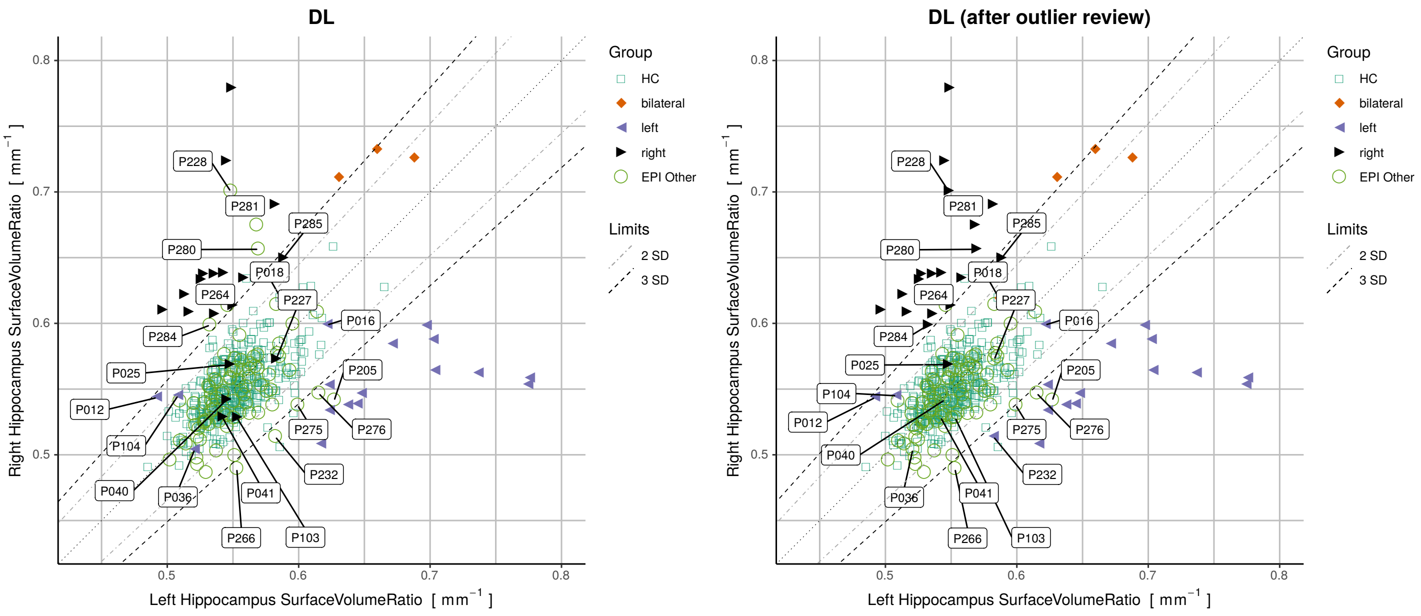

Figure S6: Surface-to-volume plot from DL with the outlier highlighted on the left and after review on the right with the revised diagnosis. Details of the reviewed changes are listed in Table S3.

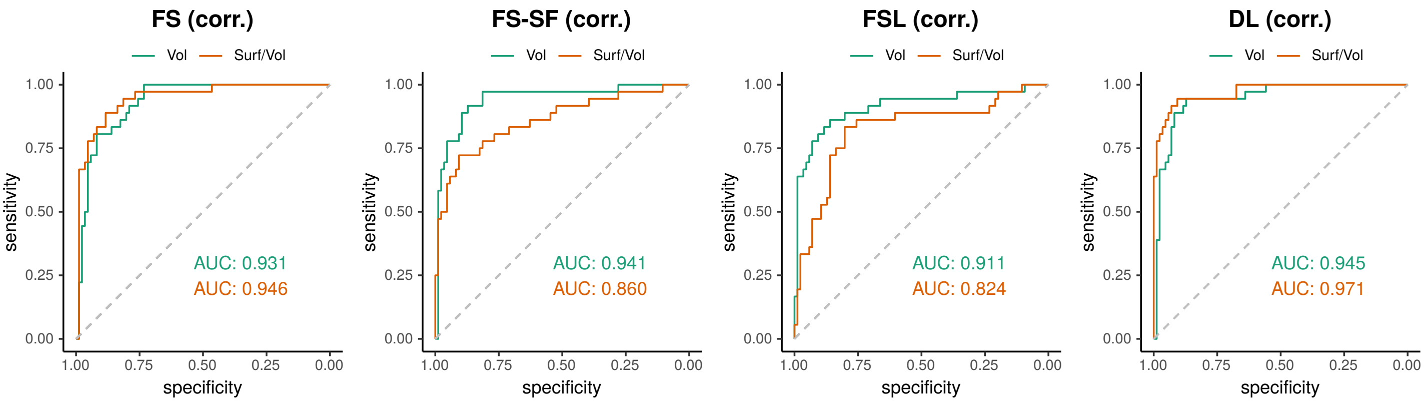

Figure S7: ROC-curves using the absolute asymmetry index (AI) to separate between HS and all-other-epilepsies **after correcting manually reviewed cases**.

5 Robustness

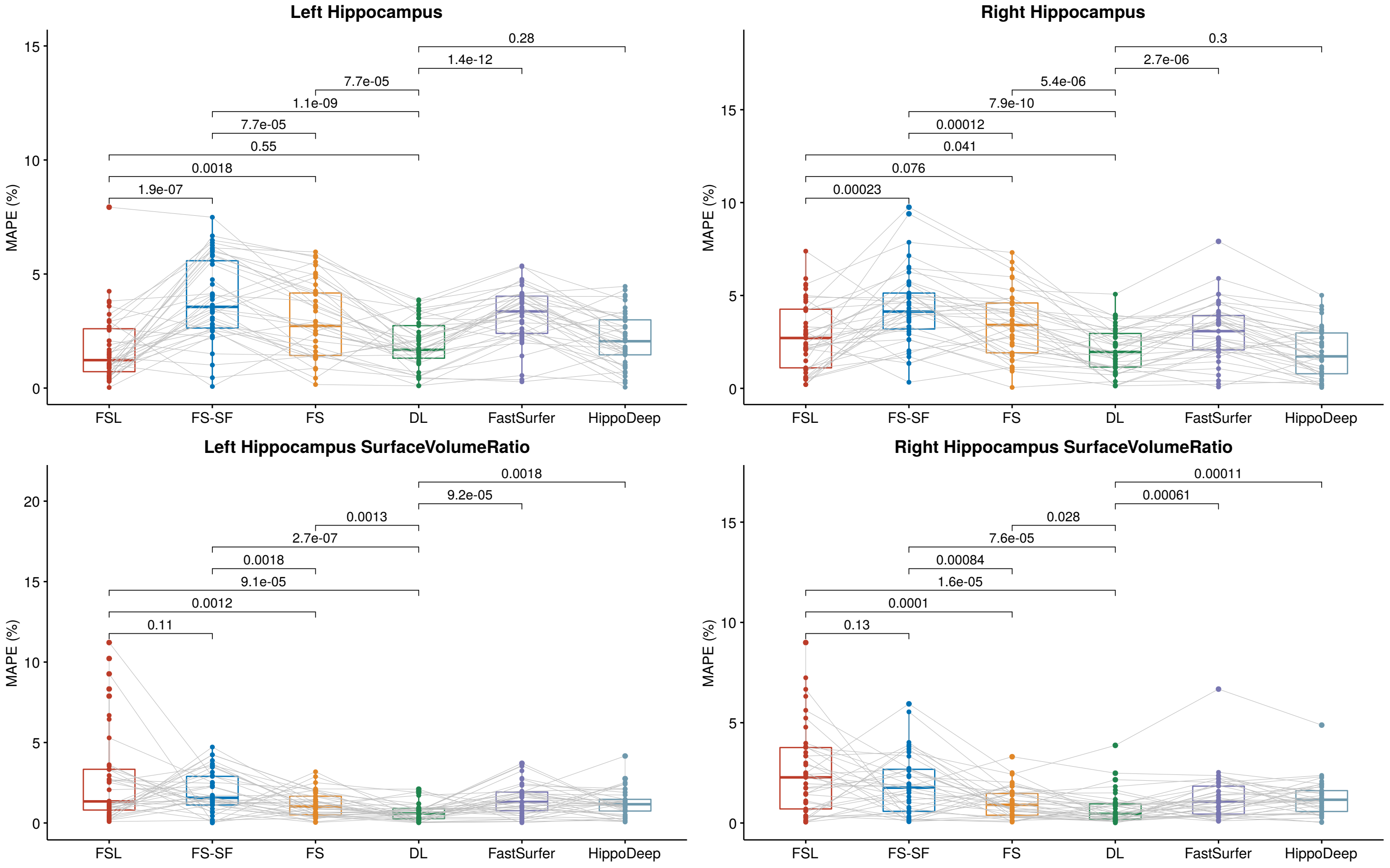

Figure S8: Robustness in terms of *mean absolute percentage error* (MAPE) evaluated on the repeated scans. Each data point represents the relative error of a session ($n = 41$). Gray lines connect corresponding sessions. p-values from a paired t-test comparing the MAPE between the methods.

6 Comparison to other DL-based Methods and Manual Tracing

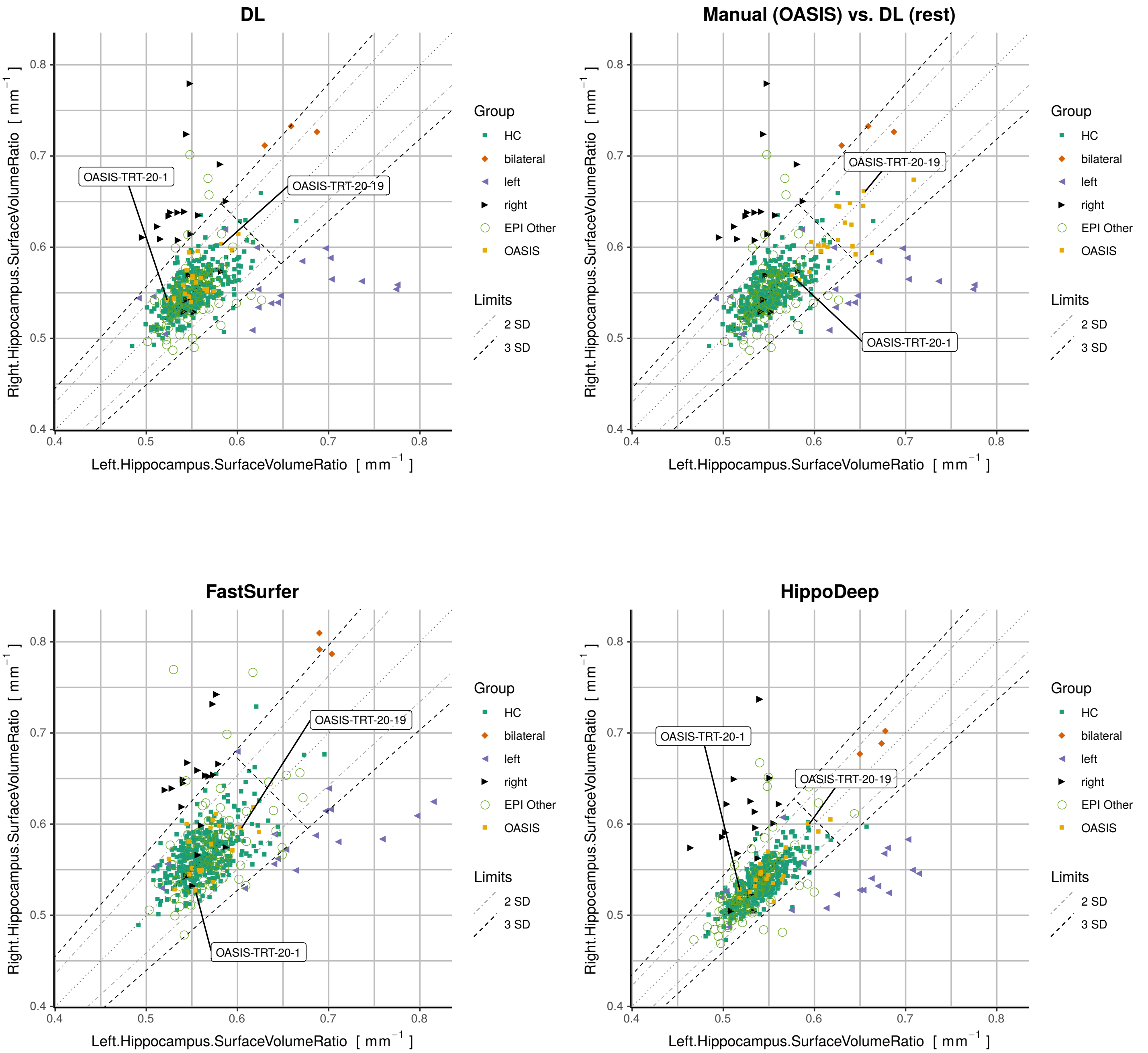

Figure S9: Plots displaying surface-to-volume ratio of left (x-axis) and right (y-axis) hippocampi with additional (healthy) subjects from the OASIS-TRT-20 dataset in yellow. In the upper right plot, the surface-to-volume ratio is derived from the manual segmentations of these cases while remaining cases (HC and patients with epilepsy) are based on the segmentations from DL+DiReCT (DL). The 2nd row shows corresponding plots with all segmentations generated with FastSurfer (left) and HippoDeep (right). Selected slices from the two hightlighted cases from OASIS can be found in the Figures below.

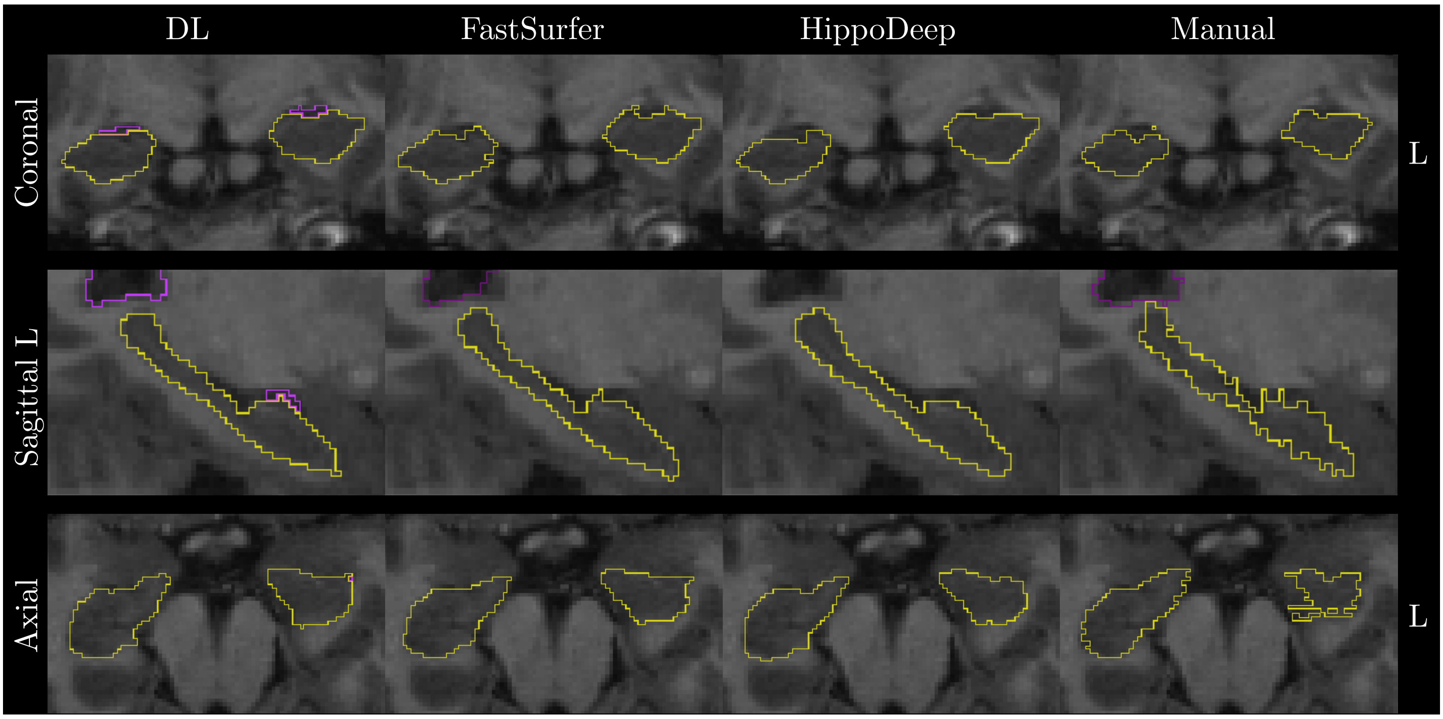

Figure S10: Qualitative example for the segmentation of the case OASIS-TRT-20-1. The case was selected as an example appearing within the cluster of the healthy controls. Images are in radiological orientation, i.e. the left (L) hemisphere appears on the right side of the image. Sagittal view shows the left hippocampus. Boundaries of the segmentation are outlined for the hippocampi (yellow) and ventricles/CSF (purple).

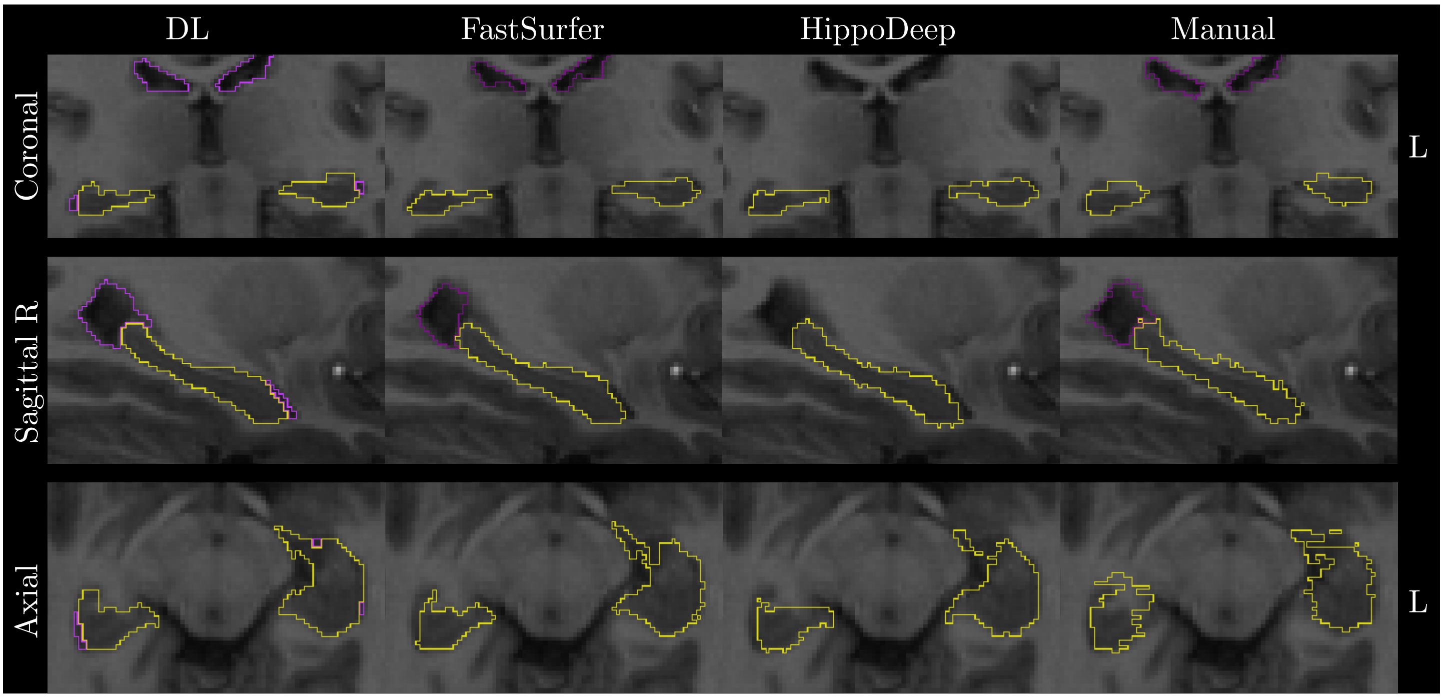

Figure S11: Qualitative example for the segmentation of the case OASIS-TRT-20-19. The case was selected as an outlier. Images are in radiological orientation, i.e. the left (L) hemisphere appears on the right side of the image. Sagittal view shows the right hippocampus. Boundaries of the segmentation are outlined for the hippocampi (yellow) and ventricles/CSF (purple).

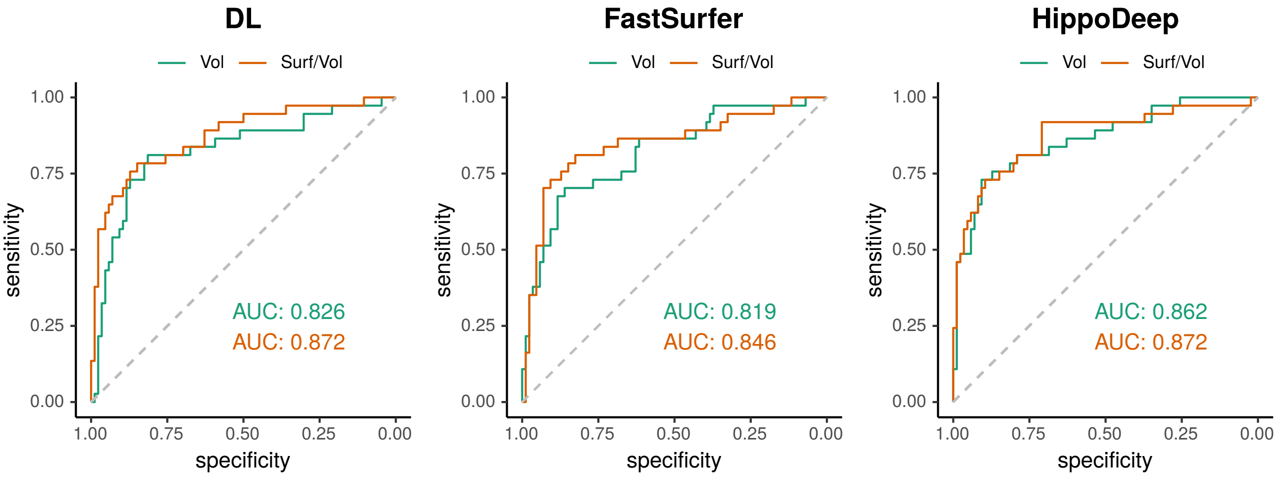

Figure S12: ROC-curves using the absolute asymmetry index (AI) to separate between HS and all-other-epilepsies contrasting results from DL+DiReCT (DL) to FastSurfer and HippoDeep. See also Figure 6 in the main text for the corresponding curves of the atlas-based methods.
